# Predictors of achieving a textbook outcome following robotic left-sided pancreatectomy: multicentre analysis

**DOI:** 10.1093/bjsopen/zraf142

**Published:** 2026-01-21

**Authors:** Abdullah K Malik, Bhargav Chikkala, Claire Ramage, Samuel J Tingle, Jason Kho, Zaed Hamady, Ali Arshad, Hassaan Bari, Andrea Sheel, Ryan Baron, Declan Dunne, Timothy Pencaval, Rajiv Lahiri, Daniel Hughes, Michael Silva, Zahir Soonawalla, Ricky Bhogal, Jeremy J French, Jose M Ramia, Jawad Ahmad, Steven A White, Sanjay Pandanaboyana, the UK Robotic Pancreatic Surgery Study Group, Abdullah K Malik, Bhargav Chikkala, Zaed Hamady, Ali Arshad, Hassaan Bari, Andrea Sheel, Ryan Baron, Declan Dunne, Timothy Pencaval, Rajiv Lahiri, Michael Silva, Zahir Soonawalla, Ricky Bhogal, Jeremy J French, Jawad Ahmad, Steven A White, Sanjay Pandanaboyana

**Affiliations:** Department of Hepato-Pancreato-Biliary and Transplant Surgery, Freeman Hospital, The Newcastle upon Tyne Hospitals NHS Foundation Trust, Newcastle upon Tyne, UK; Translational and Clinical Research Institute, Newcastle University, Newcastle upon Tyne, UK; Department of Hepato-Pancreato-Biliary and Transplant Surgery, Freeman Hospital, The Newcastle upon Tyne Hospitals NHS Foundation Trust, Newcastle upon Tyne, UK; Department of Hepato-Pancreato-Biliary and Transplant Surgery, Freeman Hospital, The Newcastle upon Tyne Hospitals NHS Foundation Trust, Newcastle upon Tyne, UK; Department of Hepato-Pancreato-Biliary and Transplant Surgery, Freeman Hospital, The Newcastle upon Tyne Hospitals NHS Foundation Trust, Newcastle upon Tyne, UK; Translational and Clinical Research Institute, Newcastle University, Newcastle upon Tyne, UK; Department of Hepato-Pancreato-Biliary Surgery, University Hospital Southampton, Southampton, UK; Department of Hepato-Pancreato-Biliary Surgery, University Hospital Southampton, Southampton, UK; Department of Hepato-Pancreato-Biliary Surgery, University Hospital Southampton, Southampton, UK; Department of Hepato-Pancreato-Biliary Surgery, University Hospitals Coventry and Warwickshire NHS Trust, Coventry, UK; Department of Pancreato-Biliary Surgery, Liverpool University Hospitals NHS Foundation Trust, Liverpool, UK; Department of Pancreato-Biliary Surgery, Liverpool University Hospitals NHS Foundation Trust, Liverpool, UK; Department of Pancreato-Biliary Surgery, Liverpool University Hospitals NHS Foundation Trust, Liverpool, UK; Department of Hepato-Pancreato-Biliary Surgery, Royal Surrey NHS Foundation Trust, Surrey, UK; Department of Hepato-Pancreato-Biliary Surgery, Royal Surrey NHS Foundation Trust, Surrey, UK; Department of Hepato-Pancreato-Biliary Surgery, Oxford University Hospitals NHS Foundation Trust, Oxford, UK; Department of Hepato-Pancreato-Biliary Surgery, Oxford University Hospitals NHS Foundation Trust, Oxford, UK; Department of Hepato-Pancreato-Biliary Surgery, Oxford University Hospitals NHS Foundation Trust, Oxford, UK; Department of Hepato-Pancreato-Biliary Surgery, The Royal Marsden NHS Foundation Trust, London, UK; Department of Hepato-Pancreato-Biliary and Transplant Surgery, Freeman Hospital, The Newcastle upon Tyne Hospitals NHS Foundation Trust, Newcastle upon Tyne, UK; Department of Surgery, Hospital General Universitario de Alicante Dr Balmis, Alicante, Spain; Department of Hepato-Pancreato-Biliary Surgery, University Hospitals Coventry and Warwickshire NHS Trust, Coventry, UK; Department of Hepato-Pancreato-Biliary and Transplant Surgery, Freeman Hospital, The Newcastle upon Tyne Hospitals NHS Foundation Trust, Newcastle upon Tyne, UK; Translational and Clinical Research Institute, Newcastle University, Newcastle upon Tyne, UK; Department of Hepato-Pancreato-Biliary and Transplant Surgery, Freeman Hospital, The Newcastle upon Tyne Hospitals NHS Foundation Trust, Newcastle upon Tyne, UK; Population Health Sciences Institute, Newcastle University, Newcastle, UK

**Keywords:** RLP, minimally invasive pancreatic surgery (MIPS), learning curve, surgical quality, postoperative pancreatic fistula (POPF), enhanced recovery after surgery (ERAS)

## Abstract

**Background:**

Recent Brescia guidelines suggest proficiency in robotic left-sided pancreatectomy (RLP) occurs after the first 21 cases (competency phase). This study reports textbook outcome (TO) rates in the competency and proficiency phases following RLP, and predictors of achieving TO.

**Methods:**

A retrospective cohort study of all RLP procedures from six UK centres was undertaken from July 2014 to August 2024. TO was defined as a composite of hospital length of stay, major morbidity, in-hospital mortality, 90-day readmission, and clinically relevant postoperative pancreatic fistula (CR-POPF). Multivariable logistic regression analysis was used to model predictors of TO.

**Results:**

In all, 281 patients underwent RLP. The median number of laparoscopic left-sided pancreatectomies undertaken before starting the RLP programme was 70 (interquartile range 40–175) per centre. In all, 109 patients underwent RLP in the competency phase and 172 underwent RLP in the proficiency phase; TO was achieved in 57 patients (52.3%) and 86 patients (50.0%), respectively (*P* = 0.801). Major morbidity occurred in 38 patients (13.5%), 68 patients were readmitted within 90 days (24.2%), and 57 patients had CR-POPF (20.3%). Patients in the proficiency phase had a longer operating time (315 *versus* 230 minutes; *P* < 0.0001), a lower rate of splenic preservation (23 *versus* 27; *P* = 0.023), and a lower rate of vascular infiltration (12 *versus* 22; *P* = 0.002) than patients in the competency phase. TO was less likely with a prolonged operation time (odds ratio 0.82 per hour; 95% c.i. 0.70 to 0.95; *P* = 0.010) with a non-linear trend noted.

**Conclusion:**

TO after RLP was achieved in half the resected patients in this UK series. There was no difference in the TO rate between the competency and proficiency phases, and previous experience with laparoscopic left-sided pancreatectomy may have contributed to this.

## Teaser

This multicentre UK study evaluated predictors of achieving a textbook outcome (TO) following robotic left-sided pancreatectomy (RLP), comparing results between the learning (competency) and proficiency phases. TO was achieved in 51.2% of patients, with no significant difference between phases. Longer operative time and male sex were associated with reduced odds of achieving a TO, whereas increasing age and higher preoperative haemoglobin were positive predictors of a TO.

## Introduction

Achieving optimal surgical outcomes is a priority in modern pancreatic surgery; as newer technologies and techniques become available, careful scrutinization is necessary to critically evaluate the safety and efficacy of novel approaches. Textbook outcome (TO) is an increasingly used metric used to define the quality of surgery delivered^1^. Broadly speaking, TO following surgery is a composite measure defined by the absence of major morbidity, readmission, prolonged length of hospital stay, and perioperative mortality, and has recently been recognized as a meaningful indicator of high-quality pancreatic surgery^2,3^.

Minimally invasive left-sided pancreatectomy (formerly distal pancreatectomy^4^) may offer potential advantages over the conventional open approach, such as reduced blood loss, shorter total length of hospital stay, and greater adherence to enhanced recovery after surgery programmes^5–9^. Recently, many pancreatic surgery programmes (including in the UK^10^), have adopted a robotic approach to left-sided pancreatectomy (RLP). The adoption of a new technology requires a structured learning curve where surgeons can develop proficiency while maintaining acceptable perioperative outcomes.

The Brescia consensus meeting concluded that the learning curve for RLP occurs in the first 21 cases for postoperative complications and 16 cases for operative time^11^. In the learning curve phase (termed the competency phase), it is recommended that surgeons engage in a structured training programme. To date, few multicentre studies have examined the factors associated with achieving a TO following RLP specifically, focusing on minimally invasive pancreatic resection in general^2,3,12^. Studies investigating the competency phase for minimally invasive pancreatic resection have largely focused on a single perioperative outcome (and are not focused on RLP)^12–15^, which may not necessarily represent the quality of surgical care. A previous multicentre study on TO after laparoscopic and open left-sided pancreatectomy (LP) has identified several variables that predict failure to achieve TO^3^, but data on RLP are lacking. TOs in the competency and proficiency phases have not been previously explored.

The present multicentre UK study aimed to compare the rates of TO during the competency and proficiency phases and to identify potential predictors of achieving a TO. A second aim was to delineate modifiable and non-modifiable factors contributing to surgical quality defined by TO.

## Methods

### Setting and population

This study was a retrospective review of all consecutive patients aged ≥ 18 years undergoing RLP for benign or malignant disease in six specialist pancreatic surgery centres in the UK between July 2014 and August 2024. No centre was declined based on volume or experience. This study is reported in line with the STROBE guidelines^16^.

Each participating centre was responsible for collecting their own anonymized data, with the local collaborator liaising with the overall study lead. The local collaborators also provided the number of laparoscopic LPs performed at each institution before starting a robotic pancreatic surgery programme.

Because this was a retrospective analysis using anonymized data, ethics approval was not required for this study, as confirmed by the UK Health Research Authority Decision tool^17^.

### Variables, definitions, and outcomes

An LP was defined as a pancreatic resection of the pancreas to the left of the superior mesenteric vein^4^. Vascular infiltration was defined as radiological or intraoperative evidence of tumour involvement of adjacent major vessels, including the splenic vein, splenic artery, or portal vein^18^. The International Study Group for Pancreatic Surgery (ISGPS) definitions for postoperative pancreatic fistula^19^, post-pancreatectomy haemorrhage^20^, and delayed gastric emptying^21^ were used. Postoperative complications were defined using the Clavien–Dindo classification^22^, and major morbidity was defined as grade IIIa or above. A TO was defined as a hospital stay during the index admission lower than the 75th percentile, no Clavien–Dindo grade IIIa or greater complications^22^, no in-hospital mortality, no conversion to open surgery, no readmission within 90 days of surgery, and no clinically relevant postoperative pancreatic fistula (ISGPS grade B/C). The competency phase was defined as the first 21 RLP cases undertaken in an individual centre, with subsequent cases being categorized as the proficiency phase based on the Brescia consensus paper^11^. During the competency phase, RLP was undertaken by two consultant surgeons operating together; therefore, during this time period, centre volume equated to surgeon volume.

Patients were classified as having undergone a multivisceral resection when another organ (excluding the spleen) was resected alongside the pancreatic specimen (either en bloc or separately). Histological diagnosis was based on postoperative pathological examination of the resected specimen by the local pathology department. Perioperative transfusion was defined as a patient receiving intraoperative or postoperative transfusion of blood products during their index admission. Operative time was defined as from the point of incision up to the point of wound closure and application of dressings. Patients were classified as having undergone conversion to an open procedure if any aspect of the dissection or resection was performed through an open wound (other than extraction of the resected specimen).

### Statistical analyses

Descriptive statistics were used, with continuous data presented as the median with interquartile range (i.q.r.) and categorical data presented as frequencies and percentages. The significance of differences between groups was analysed using the χ^2^ test or Fisher’s exact test for categorical data, and the Mann–Whitney *U* test or *t* test for continuous data. Missing data were addressed using multiple imputation by chained equations, with five imputed data sets generated. Results from the five imputed data sets were pooled using Rubin’s rules, and analysed.

Predictors of a TO were identified by selecting potential patient-level variables previously identified to affect perioperative outcome *a priori*, and fitting them into a multivariable mixed-effects logistic regression model, treating centre as a random effect. This approach allows for adjustment of differences in patient case-mix and centre-level effects that could influence outcomes. The initial model included operative time, but did not include factors that were likely to increase operative time (splenic preservation, multivisceral resection, and conversion to open) to avoid collinearity. A sensitivity analysis was performed, removing operative time and fitting the latter factors into the model.

The logistic regression analysis was further repeated for TO using restricted cubic splines with three knots at the 10th, 50th and 90th percentiles to model the potential non-linear relationship between operative time and TO. Pooled data were used for this analysis as well. The resulting model produced estimates of the adjusted odds ratio (OR) and 95% confidence interval (c.i.) for achieving a TO across the range of operation times in the data set. The reference point was defined as the median operating time where the OR was set at 1. Wald χ^2^ testing was used to assess the overall and non-linear contributions of each variable to the model.

To determine whether any identified potential relationships in the entire cohort were dependent on whether patients were operated on during the competency phase or proficiency phase, multivariable modelling was repeated with cases performed during the learning curve excluded.

To assess for between-centre variation, a funnel plot was produced for the observed/expected (O/E) ratio for achieving a TO following RLP by centre volume. Expected probabilities were derived from the mixed-effects logistic regression model. Observed and expected outcomes were aggregated by centre, and O/E ratios were calculated for each centre. An O/E ratio > 1 indicates that the centre had better TO rates than expected based on its case mix, whereas an O/E ratio < 1 indicates worse TO rates than expected. To assess variability, smooth 95% and 99.8% control limits were calculated as continuous functions of centre size using standard errors. Centres were plotted by their volume against the O/E ratio, with any centres falling outside of the control limits considered as potential outliers.

For all statistical tests, significance was two-tailed and set at *P* < 0.05. All analyses were performed using R (R Foundation for Statistical Computing, Vienna, Austria).

## Results

### Baseline patient characteristics and procedures

In all, 281 patients were included in this analysis, with a median age of 63 (i.q.r. 55–73) years; 153 patients (54.4%) were female. Of the 281 patients in the study, 109 (38.8%) were allocated to the competency phase. Most patients had an American Society of Anaesthesiologists (ASA) grade of II or III (255, 90.7%) with a neuroendocrine tumour as the most common histology (91, 32.4%). Splenic preservation was performed in 47 patients (16.7%) and 30 patients (10.7%) underwent multivisceral resection. Conversion to an open procedure occurred in 23 patients (8.2%) and the median operative time was 281 (i.q.r. 200–360) minutes (min). Drains were placed at the time of operation in 276 patients (98.2%) along the pancreatic transection margin, and 97 patients (35.1%) were discharged from hospital with a drain in place. The median time to drain removal in the cohort was 6 (i.q.r. 4–11) days.

*Table 1* presents baseline characteristics for the entire cohort, as well as for patients in the competency and proficiency phases separately. Patients in the proficiency phase had a longer operating time (315 *versus* 230 min; *P* < 0.0001), a lower rate of splenic preservation (23 (13.4%) *versus* 27 (24.8%); *P* = 0.023), and a lower rate of vascular infiltration (12 (7.0%) *versus* 22 (20.2%); *P* = 0.002).

**Table 1:** Baseline characteristics of all patients undergoing robotic left-sided pancreatectomy and of the competency and proficiency cohorts separately

	Whole cohort (*n* = 281)	Competency phase (*n* = 109)	Proficiency phase (*n* *=* 172)	P*
Age (years), median (i.q.r.)	63 (55–73)	63 (56–72)	64 (55–74)	0.696
**Sex**				0.078
Female	153 (54.4%)	67 (61.5%)	86 (50.0%)	
Male	128 (45.6%)	42 (38.5%)	86 (50.0%)	
BMI (kg/m^2^), median (i.q.r.)	26.2 (23.3–30.9)	25.7 (23.1–31.0)	26.7 (23.6–30.6)	0.474
**ASA grade**				0.194
I	25 (8.9%)	12 (11.0%)	13 (7.6%)	
II	170 (60.5%)	70 (64.2%)	100 (58.1%)	
III	86 (30.6%)	27 (24.8%)	59 (34.3%)	
Previous abdominal surgery	48 (17.1%)	24 (22.0%)	24 (14.0%)	0.112
Diabetes	82 (29.2%)	36 (33.0%)	46 (26.7%)	0.320
Previous MI	23 (8.2%)	8 (7.3%)	15 (8.7%)	0.851
Previous CVA	17 (6.0%)	6 (5.5%)	11 (6.4%)	0.961
Previous pancreatitis	23 (8.2%)	9 (8.3%)	14 (8.1%)	1.00
**Histological diagnosis**				0.363
Neuroendocrine tumour	91 (32.4%)	39 (35.8%)	52 (30.2%)	
Adenocarcinoma	62 (22.1%)	21 19.3%)	42 (24.4%)	
IPMN	38 (13.5%)	18 (16.5%)	20 (11.6%)	
Other	90 (32.0%)	31 (28.4%)	58 (33.7%)	
Vascular infiltration	34 (12.1%)	22 (20.2%)	12 (7.0%)	0.002
Converted to open	23 (8.2%)	10 (9.2%)	13 (7.6%)	0.796
Operative time (min), median (i.q.r.)	281 (200–360)	230 (180–315)	315 (240–400)	<0.0001
Splenic preservation	50 (17.8%)	27 (24.8%)	23 (13.4%)	0.023
Multivisceral resection	30 (10.7%)	8 (7.3%)	22 (12.8%)	0.214

Values are *n* (%) unless otherwise stated. i.q.r., interquartile range; BMI, body mass index; ASA, American Society of Anesthesiologists; MI, myocardial infarction; CVA, cerebrovascular accident; IPMN, intraductal papillary mucinous neoplasm; min, minutes. *Differences between groups were analysed using the *χ*^2^ test or Fishers’ exact test for categorical data, and the Mann–Whitney *U* test or *t*-test for continuous data.

### Textbook outcomes

A TO was achieved in 143 patients (50.9%) across the entire cohort, and was similar in the competency and proficiency phases (52.3% *versus* 50.0%, respectively; *P* = 0.801). A comparison of outcomes in the competency and proficiency phase is presented in *Table 2*. The median length of hospital stay for the entire cohort was 7 (i.q.r. 5–9) days. Stratified by phase, the median length of hospital stay was shorter in the proficiency group than in the competency group (6 (i.q.r. 5–8) *versus* 7 (i.q.r. 5–10) days, respectively; *P* = 0.032). Sixty-five patients (23.1%) were readmitted within 90 days of surgery. Seventeen patients (6.0%) experienced a Clavien–Dindo grade IIIa complication or higher within 90 days of surgery (2 in-hospital deaths, 0.7%) and 16 patients (5.7%) required a perioperative transfusion. Fifty-six patients (19.9%) developed a clinically relevant postoperative pancreatic fistula, with 8 (2.8%) and 12 (4.3%) developing a postpancreatectomy haemorrhage and delayed gastric emptying, respectively. Three patients (1.1%) underwent further surgery for postoperative complications, all of whom were in the competency phase.

**Table 2:** Outcomes following robotic left-sided pancreatectomy in the entire cohort and in the competency phase and proficiency phase separately

	Whole cohort (*n* = 281)	Competency phase (*n* = 109)	Proficiency phase (*n* = 172)	P*
LOS (days), median (i.q.r.)	7 (5–9)	6 (5–8)	7 (5–10)	0.032
Hospital stay < 75th percentile	192 (68.3%)	82 (75.2%)	110 (64.0%)	0.065
Readmission	68 (24.2%)	30 (27.5%)	38 (22.1%)	0.372
CR-POPF	57 (20.3%)	25 (22.9%)	32 (18.6%)	0.467
CD grade ≥ IIIa complication	38 (13.5%)	17 (15.6%)	21 (12.2%)	0.529
Perioperative transfusion	16 (5.7%)	6 (5.5%)	10 (3.6%)	1.00
Reoperation	3 (1.1%)	3 (2.8%)	0 (0.0%)	0.113
In-hospital mortality	2 (0.7%)	1 (0.4%)	1 (0.4%)	1.00
Textbook outcome	143 (50.8%)	57 (52.3%)	86 (50.0%)	0.801

Values are *n* (%) unless otherwise stated. LOS, length of hospital stay; i.q.r., interquartile range. CR-POPF, clinically relevant postoperative pancreatic fistula; CD, Clavien–Dindo. *Differences between groups were analysed using the *χ*^2^ test or Fishers’ exact test for categorical data, and the Mann–Whitney *U* test or *t*-test for continuous data.

### Predictors of achieving a TO

The multivariable mixed-effects logistic regression model is presented in *Table 3*. Increasing age (OR 1.03 per year; 95% c.i. 1.01 to 1.05; *P* = 0.003) and preoperative haemoglobin (OR 1.03 per g/L; 95% c.i. 1.01 to 1.05; *P* = 0.006) were identified as significant predictors of achieving a TO. Male sex was detrimental to achieving a TO (OR 0.52; 95% c.i. 0.28 to 0.97; *P* = 0.039). Preoperative pancreatitis predicted failure to achieve a TO following RLP (OR 0.26; 95% c.i. 0.09 to 0.78; *P* = 0.016). Operative time was identified as a significant predictor (OR 0.82 per hour; 95% c.i. 0.70 to 0.95; *P* = 0.007), with an 18% reduction in the odds of achieving a TO for every additional hour.

**Table 3:** Multivariable mixed-effects logistic regression model identifying potential predictors for achieving a textbook outcome following robotic left-sided pancreatectomy

	Odds ratio	*P*
Age (per year)	1.03 (1.01, 1.05)	0.003
Sex (male *versus* female)	0.52 (0.28, 0.97)	0.039
BMI (per kg/m^2^)	0.96 (0.92, 1.01)	0.147
**Histological diagnosis**		0.443
Neuroendocrine tumour	Reference	
Adenocarcinoma	0.89 (0.40, 1.98)	0.783
Intraductal papillary mucinous neoplasm	1.71 (0.72, 4.03)	0.221
Other	1.37 (0.70, 2.66)	0.358
Vascular infiltration	1.56 (0.65, 3.77)	0.323
Previous major abdominal surgery	0.92 (0.0.44, 1.94)	0.828
Preoperative haemoglobin (per g/L)	1.03 (1.01, 1.05)	0.006
Previous myocardial infarction	0.96 (0.36, 2.55)	0.942
Previous cerebrovascular accident	0.55 (0.18, 1.74)	0.310
Diabetes	1.38 (0.76, 2.51)	0.294
Previous pancreatitis	0.26 (0.09, 0.78)	0.016
Operative time (per hour)	0.82 (0.70, 0.95)	0.010

Values in parentheses are 95% confidence intervals. BMI, body mass index.

A sensitivity analysis was performed removing operative time from the model and inserting two factors that could be associated with a prolonged operation (splenic preservation and multivisceral resection). Age, sex, preoperative haemoglobin, and preoperative pancreatitis remained as significant predictors of achieving a TO, with similar effects as in the initial model (*Table S1*).

### Restricted cubic spline modelling and operative time

**Fig. 1:**
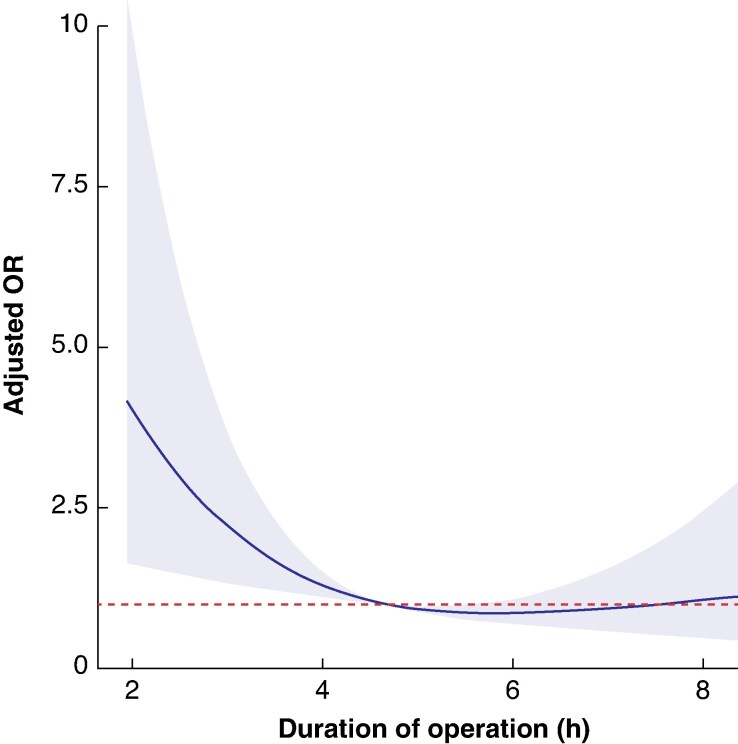
Restricted cubic spline showing the adjusted OR for achieving a textbook outcome as a function of operative time The dashed red line represents the reference (adjusted OR of 1). The shaded area represents the 95% confidence interval. OR, odds ratio; h, hours. Test of significance, *P*  *=* 0.005; test of non-linearity, *P*  *=* 0.032.

To further delineate the potential relationship between operative time and the adjusted OR for achieving a TO, a restricted cubic spline was plotted (*Fig. 1*). This showed a non-linear relationship and confirmed that increasing operative time was significantly associated with a falling adjusted OR for achieving a TO (test of non-linearity *P* = 0.032). Patients undergoing RLP were more likely to achieve a TO where the operative time was < 4.68 hours (281 min), with patients exceeding this threshold less likely to achieve a TO. Beyond 6 hours, the confidence interval crosses 1, suggesting that for very prolonged operative times the precise impact on TO is uncertain.

### Subanalysis of cases during the proficiency phase

Age, preoperative haemoglobin, and preoperative pancreatitis were retained as predictors of TO when cases performed during the competency phase were excluded from the main model, with similar effect sizes (*Table S2*). On multivariable modelling, operation time and male sex were no longer predictors of TO. To explore the potential relationship between operating time and TO in the proficiency phase, a restricted cubic spline was plotted (*Fig. S1*), showing a similar trend to the full cohort, although statistical significance was not reached for overall effect (*P*  *=* 0.088) or non-linearity (*P* = 0.142).

### Between-centre variation

During the study period, the median total case volume for RLP in the six centres was 36 (i.q.r. 35–38); one centre performed 126 RLP as the maximum case volume in the cohort and another performed 11 as the minimum case volume. The median annual case volume for the whole study period was 9 (i.q.r. 6–11) cases per year. From 2014 to 2019, there were two centres performing a median of six and nine RLPs per year each; from 2019 onwards, the median annual volume for all six centres was 11 (i.q.r. 6–13) RLPs per year.

**Fig. 2:**
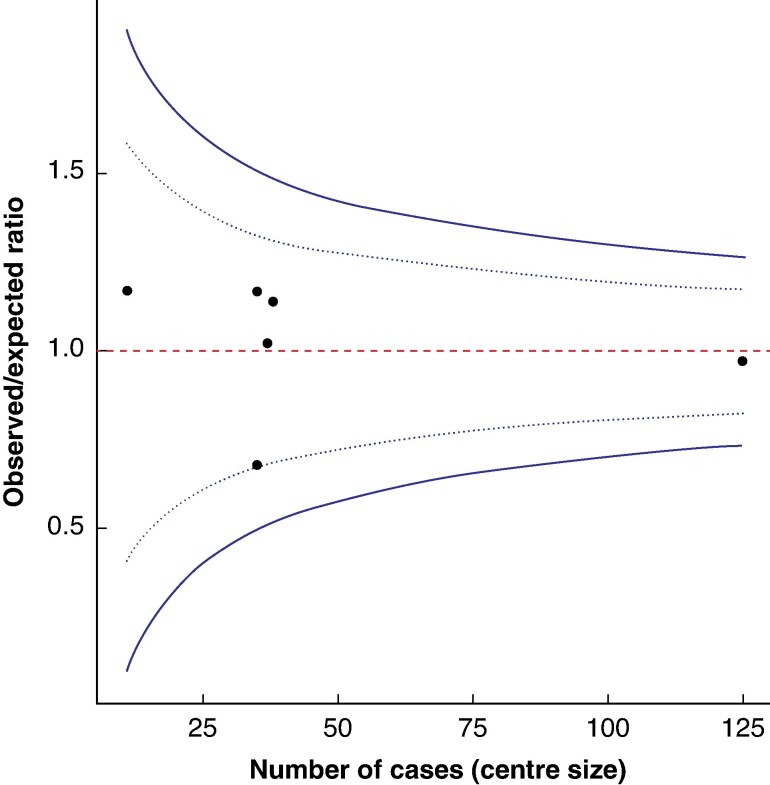
Funnel plot of between-centre variation in textbook outcomes following robotic left-sided pancreatectomy, showing the observed/expected ratio by centre volume The dashed red line represents the reference point where the observed/expected ratio is equal to 1.0. The dotted and solid blue lines indicate the 95% and 99.8% control limits, respectively. Symbols indicate individual centre observed/expected ratios.

Before initiating a robotic pancreatic surgery programme, the median number of laparoscopic LPs performed was 70 (i.q.r. 40–175). For RLP, the unadjusted TO rate ranged from 34.3 to 81.8%; when adjusting for case mix using the logistic regression model, the TO O/E ratio ranged from 0.68 to 1.17 per centre. No centres were classed as outliers in the funnel plot, with centres generally clustering around an O/E ratio of 1.0, suggesting that TO was not explicitly dependent on centre volume (*Fig. 2*).

## Discussion

This study evaluated the outcomes and characteristics of patients achieving a TO following RLP. This analysis highlighted key insights into patient selection, operative factors, and their implications for surgical success, as defined by achieving a TO, following RLP. This was the first analysis specifically investigating predictors of TO for RLP, with other studies including other types of pancreatic resection, laparoscopic procedures and liver/biliary procedures^2,3,12,23–25^.

The rate of patients achieving a TO following RLP was similar in the competency and proficiency phases. Key differences in operative variables when comparing the competency phase with the proficiency phase were a significantly longer operating time, lower rate of splenic preservation, and a lower rate of vascular infiltration in the proficiency phase. This may represent a greater degree of complexity in case selection during the proficiency phase compared with the learning curve (competency) phase, which is not objectively measurable. Some surgeons or centres may have undertaken more complex cases before surpassing the competency phase, but this was not assessed in the present study. Achieving similar TO rates in the competency and proficiency phases may suggest that increasing case complexity once proficiency is achieved is not detrimental to patient outcome.

Studies on the competency phase in robotic pancreatic surgery are relatively heterogeneous, with many series focusing on robotic pancreatoduodenectomy (or including it with RLP cases) or reporting learning curves for minimally invasive pancreatic surgery in general^12,13,26–29^. The only study exploring TO during the competency phase for RLP investigated minimally invasive LP in 2610 patients, including 349 robotic procedures, with an overall TO rate of 65.8%^12^. Lof *et al*.^12^ reported a breakpoint of 85 minimally invasive procedures for the competency phase with respect to achieving a stable TO rate of 70%, and 56 procedures for reducing operative time, both well above the 21 cases recommended by the Brescia guidelines for morbidity rates^11^. This may be due, in part, to TO being a composite measure, with individual components influenced by many factors. As a multicentre study across a variety of settings in Europe, cultural differences, practice variations, and other unknown confounders may have affected the results of the study^12^. Although the 21-case threshold defines proficiency in the Brescia guidelines, in some centres this was not reached on an individual surgeon level. On initiating a robotic pancreatic surgery programme, each unit identified two surgeons who were supervised by a robotic pancreatic surgery proctor; therefore, surgeon volume will have closely approximated centre volume.

On logistic regression modelling, every 1-hour increase in operating time was associated with an 18% reduction in the odds of achieving a TO. The precise mechanism by which operative time affects outcome is unknown, but is likely related to the degree of case complexity. This analysis suggests that with increasing operative time, the likelihood of achieving a TO falls until it reaches a threshold where operative time no longer has an effect.

TO rates varied between centres from 34.3 to 81.8%, although most centres were found to cluster around the O/E ratio of 1 when adjusting for case mix, suggesting that good outcomes can be achieved independent of robotic case volume. The established laparoscopic programmes in each centre are likely to have contributed to this observation. Furthermore, all centres were based in the UK and have established enhanced recovery after surgery programmes, which may have contributed to the standardization of outcomes. Although the overall TO rate in the present study is lower than other published series^2,3,24,25^, this may be due, in part, to half the cases in this cohort being performed within the competency phase.

A recent study investigated predictors of TO in 450 patients undergoing LP in eight high-volume centres^3^. The authors reported an overall TO rate of 58.2%. In that study, only delayed gastric emptying and postoperative pancreatic fistula were identified as predictors of failing to achieve TO^3^. The main limitation in that study’s design is that both delayed gastric emptying and postoperative pancreatic fistula are both complications rather than baseline predictors, resulting in collider bias within the model. The authors did not adjust for potential nesting of cases, further impacting model validity^3^.

The results of the present study should be interpreted within the context of its limitations. This was a retrospective analysis and there is likely to have been a degree of selection bias potentially affecting the results. Although TO is a binary metric, it is composed of variables that can be significantly influenced by centre variations (for example, practices related to postoperative pancreatic fistula management). Although a mixed-effects regression model with centre as a random effect was used, the model does not eliminate systematic differences in care protocols, resourcing or surgical techniques. Length of hospital stay was a component of TO because it provides a potential surrogate for postoperative costs, although it could be influenced by multiple confounders, such as institutional practices and differing postoperative care protocols. There were similar trends in the sensitivity analysis excluding competency phase procedures; however, the reduced sample size could have masked the impact of potential predictors resulting in a type 2 error.

Although TO is one metric for surgical quality, oncological endpoints, such as resection margin and lymph node yield, were not assessed in this analysis because a significant proportion of patients will undergo LP for benign conditions.

RLP were not compared with open or laparoscopic approaches because this was beyond the scope of the study. Although a learning curve by discrete phases based on case number was defined, this is a surrogate for a true learning curve, which typically requires surgeon-level data and cumulative sum analysis. Given the multicentre design and multiple surgeons per centre, this was not feasible but should be explored in future studies.

Despite these limitations, the present study presents the first detailed analysis of predictors of achieving a TO following RLP and could aid future patient selection for new centres establishing programmes. Although the predictors of TO identified in this study are not novel, the consistency of these findings with established surgical predictors across different procedures supports the external validity.

## Collaborators

The members of the UK Robotic Pancreatic Surgery Study group are: Abdullah K. Malik (Newcastle upon Tyne Hospitals NHS Foundation Trust, Newcastle upon Tyne, UK); Bhargav Chikkala (Newcastle upon Tyne Hospitals NHS Foundation Trust, Newcastle upon Tyne, UK); Zaed Hamady (University Hospital Southampton, Southampton, UK); Ali Arshad (University Hospital Southampton, Southampton, UK); Hassaan Bari (University Hospitals Coventry and Warwickshire NHS Trust, Coventry, UK); Andrea Sheel (Liverpool University Hospitals NHS Foundation Trust, Liverpool, UK); Ryan Baron (Liverpool University Hospitals NHS Foundation Trust, Liverpool, UK); Declan Dunne (Liverpool University Hospitals NHS Foundation Trust, Liverpool, UK); Timothy Pencaval (Royal Surrey NHS Foundation Trust, Guildford, UK); Rajiv Lahiri (Royal Surrey NHS Foundation Trust, Guildford, UK); Michael Silva (Oxford University Hospitals NHS Foundation Trust, Oxford, UK); Zahir Soonawalla (Oxford University Hospitals NHS Foundation Trust, Oxford, UK); Ricky Bhogal (The Royal Marsden NHS Foundation Trust, London, UK); Jeremy J. French (Newcastle upon Tyne Hospitals NHS Foundation Trust, Newcastle upon Tyne, UK); Jawad Ahmad (University Hospitals Coventry and Warwickshire NHS Trust, Coventry, UK); Steven A. White (Newcastle upon Tyne Hospitals NHS Foundation Trust, Newcastle upon Tyne, UK); and Sanjay Pandanaboyana (Newcastle upon Tyne Hospitals NHS Foundation Trust, Newcastle upon Tyne, UK).

## Supplementary Material

zraf142_Supplementary_Data

## Data Availability

Anonymized data may be made available upon request at the discretion of the corresponding author and centre leads.
