# Supplementary material for: Predictors of achieving a textbook outcome following robotic left-sided pancreatectomy: multicentre analysis
Source: BJS Open. 2026 Jan 21;10(1):zraf142. doi: 10.1093/bjsopen/zraf142 (PMC12822603; doi:10.1093/bjsopen/zraf142)
Supplement: zraf142_Supplementary_Data [file zraf142_supplementary_data.docx]

**Predictors of achieving a textbook outcome following robotic left-sided pancreatectomy: multicenter analysis**

Abdullah K. Malik^1,2^, Bhargav Chikkala^1^, Claire Ramage^1^, Samuel J. Tingle^1,2^, Jason Kho^3^, Zaed Hamady^3^, Ali Arshad^3^, Hassaan Bari^4^, Andrea Sheel^5^, Ryan Baron^5^, Declan Dunne^5^, Timothy Pencaval^6^, Rajiv Lahiri^6^, Daniel Hughes^7^, Michael Silva^7^, Zahir Soonawalla^7^, Ricky Bhogal^8^, Jeremy J. French^1^, Jose M. Ramia^9^, Jawad Ahmad^4^, Steven A. White^1,2^, Sanjay Pandanaboyana^1,10^

*On behalf of the UK Robotic Pancreatic Surgery Study Group (UKROPS Group)*

1 – Department of HPB and Transplant Surgery, Freeman Hospital, The Newcastle upon Tyne Hospitals NHS Foundation Trust, Newcastle upon Tyne, UK

2 – Translational and Clinical Research Institute, Newcastle University, Newcastle upon Tyne, UK

3 – Department of HPB Surgery, University Hospital Southampton, Southampton, UK

4 – Department of HPB Surgery, University Hospitals Coventry and Warwickshire NHS Trust, Coventry, UK

5 – Department of Pancreato-biliary Surgery, Liverpool University Hospitals NHS Foundation Trust, Liverpool, UK

6 – Department of HPB Surgery, Royal Surrey NHS Foundation Trust, Surrey, UK

7 – Department of HPB Surgery, Oxford University Hospitals NHS Foundation Trust, Oxford, UK

8 – Department of HPB Surgery, The Royal Marsden NHS Foundation Trust, London, UK

9 – Department of Surgery, Hospital General Universitario de Alicante Dr Balmis, Alicante, Spain

10 - Population Health Sciences Institute, Newcastle University, Newcastle, UK

**Corresponding author:**

Professor Sanjay Pandanaboyana, FRCS

Consultant HPB Surgeon

Freeman hospital

Newcastle Upon Tyne

Email: Sanjay.pandanaboyana@ncl.ac.uk

Twitter: @Sanjay_HPB

**Supplementary Materials - Index**

| **Supplementary Figures and Tables** |  |
| --- | --- |
| Supplementary figure 1 | *pag. 2* |
| Supplementary table 1 | *pag. 3* |
| Supplementary table 2 | *pag. 4* |

**Supplementary Figures and Tables**


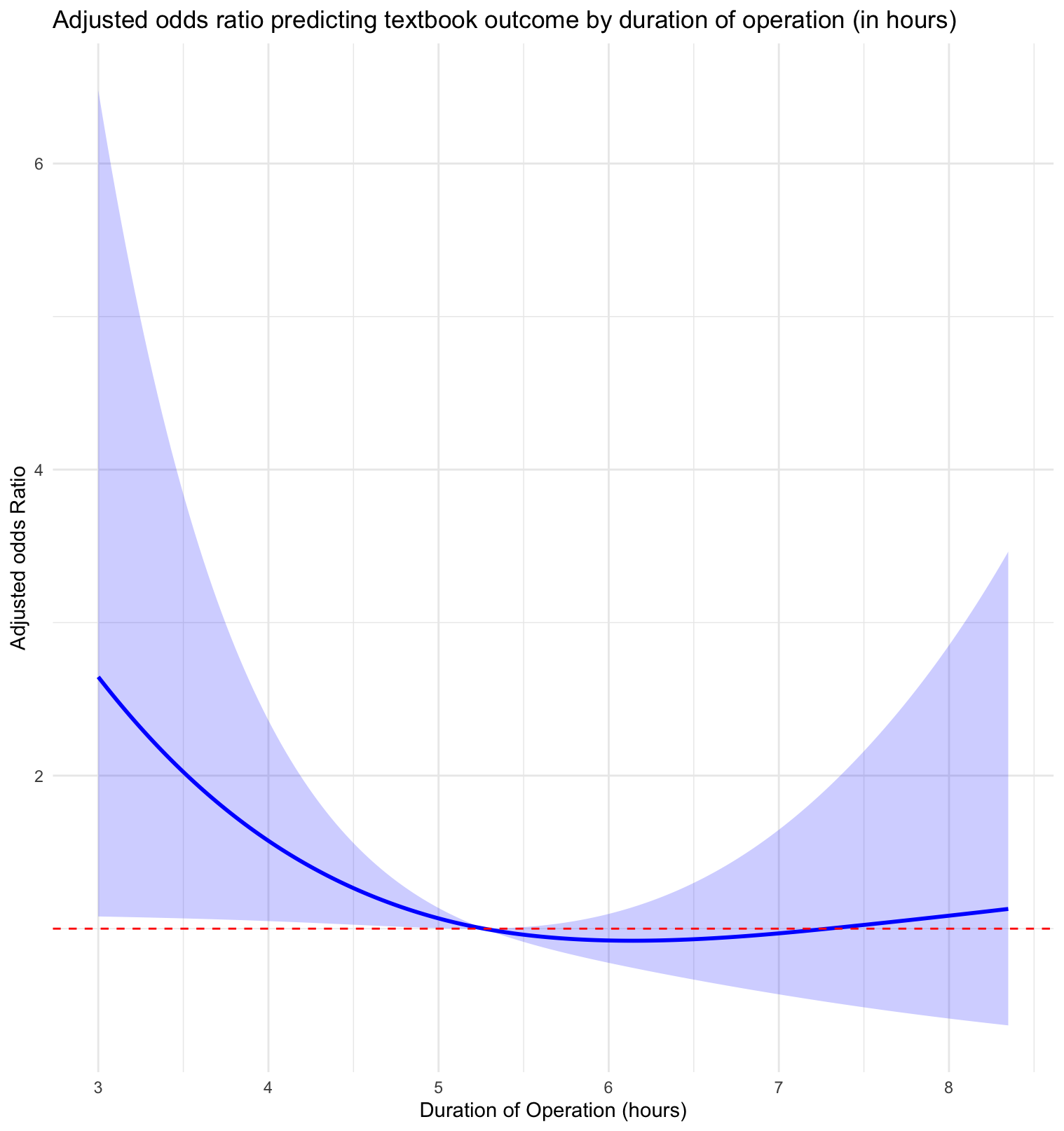
 Figure S1 – Restricted cubic spline displaying the adjusted odds ratio for achieving a textbook outcome as a function of operative time (test for significance *P*=0.088, test for non-linearity *p*=0.142) in proficiency phase cases, with the dashed red line representing the reference (adjusted odds ratio of 1). The restricted cubic spline had 3 knots and was adjusted for all factors.

Table S1 - Multivariable logistic regression for textbook outcome excluding operating time, but including potential predictors of a longer operation

| **Variable** | **OR (95% CI)** | **P-value** |
| --- | --- | --- |
| Age (per year) | 1.04 (1.02-1.06) | 0.001 |
| Sex (male versus female) | 0.44 (0.24-0.82) | 0.010 |
| BMI (per kg/m^2^) | 0.96 (0.91-1.01) | 0.087 |
| Histological diagnosis  *Neuroendocrine tumour*  *Adenocarcinoma*  *Intraductal papillary mucinous neoplasm*  *Other* | -  (ref)  0.79 (0.36-1.77)  1.39 (0.59-3.30)  1.53 (0.76-3.06) | 0.371  (ref)  0.573  0.451  0.234 |
| Vascular infiltration | 1.28 (0.45-3.70) | 0.644 |
| Preoperative haemoglobin (per g/L) | 1.03 (1.01-1.05) | 0.005 |
| Previous myocardial infarction | 0.80 (0.30-2.13) | 0.657 |
| Previous cerebrovascular accident | 0.64 (0.20-2.06) | 0.458 |
| Diabetes mellitus | 1.47 (0.80-2.71) | 0.219 |
| Previous pancreatitis | 0.23 (0.07-0.73) | 0.013 |
| Splenic preservation | 1.83 (0.86-3.86) | 0.115 |
| Multi-visceral resection | 0.65 (0.27-1.57) | 0.333 |

Table S2 - Multivariable mixed effects logistic regression model identifying potential predictors for achieving a textbook outcome following robotic left sided pancreatectomy in proficiency phase cases only

| **Variable** | **OR (95% CI)** | **P-value** |
| --- | --- | --- |
| Age (per year) | 1.03 (1.01-1.06) | 0.011 |
| Sex (male versus female) | 0.59 (0.25-1.35) | 0.209 |
| BMI (kg/m^2^) | 0.98 (0.92-1.04) | 0.484 |
| Histological diagnosis  *Neuroendocrine tumour*  *Adenocarcinoma*  *Intraductal papillary mucinous neoplasm*  *Other* | -  (ref)  0.53 (0.17-1.63)  1.28 (0.39-4.21)  1.35 (0.56-3.28) | 0.444  (ref)  0.266  0.683  0.504 |
| Vascular infiltration | 1.35 (0.25-7.41) | 0.727 |
| Previous major abdominal surgery | 0.65 (0.23-1.87) | 0.425 |
| Preoperative haemoglobin (per g/L) | 1.03 (1.00-1.05) | 0.019 |
| Previous myocardial infarction | 0.53 (0.14-1.92) | 0.330 |
| Previous cerebrovascular accident | 0.86 (0.17-4.25) | 0.851 |
| Diabetes mellitus | 0.98 (0.42-2.26) | 0.954 |
| Previous pancreatitis | 0.15 (0.03-0.81) | 0.027 |
| Operative time (per hour) | 0.87 (0.68-1.12) | 0.279 |
